# Both Diet and Sociality Affect Primate Brain-Size Evolution

**DOI:** 10.1093/sysbio/syac075

**Published:** 2022-12-01

**Authors:** Mark Grabowski, Bjørn T Kopperud, Masahito Tsuboi, Thomas F Hansen

**Affiliations:** Research Centre in Evolutionary Anthropology and Palaeoecology, Liverpool John Moores University, 3 Byrom Street, Liverpool L3 3AF, UK; Department of Biosciences, University of Oslo, Blindernveien 31, 0371 Oslo, Norway; GeoBio-Center LMU, Ludwig-Maximilians-Universität München, Richard-Wagner Straße 10, 80333 Munich, Germany; Department of Earth and Environmental Sciences, Paleontology and Geobiology, Ludwig-Maximilians-Universität München, Richard-Wagner Straße 10, 80333 Munich, Germany; Department of Biosciences, University of Oslo, Blindernveien 31, 0371 Oslo, Norway; Department of Biology, Lund University, Ekologihuset, Sölvegatan 37, 223 62 Lund, Sweden; Department of Biosciences, University of Oslo, Blindernveien 31, 0371 Oslo, Norway

## Abstract

Increased brain size in humans and other primates is hypothesized to confer cognitive benefits but brings costs associated with growing and maintaining energetically expensive neural tissue. Previous studies have argued that changes in either diet or levels of sociality led to shifts in brain size, but results were equivocal. Here we test these hypotheses using phylogenetic comparative methods designed to jointly account for and estimate the effects of adaptation and phylogeny. Using the largest current sample of primate brain and body sizes with observation error, complemented by newly compiled diet and sociality data, we show that both diet and sociality have influenced the evolution of brain size. Shifting from simple to more complex levels of sociality resulted in relatively larger brains, while shifting to a more folivorous diet led to relatively smaller brains. While our results support the role of sociality, they modify a range of ecological hypotheses centered on the importance of frugivory, and instead indicate that digestive costs associated with increased folivory may have resulted in relatively smaller brains. [adaptation; allometry; *bayou*; evolutionary trend; energetic constraints; phylogenetic comparative methods; primate brain size; *Slouch*; social-brain hypothesis.]

Brain size varies greatly among primates (e.g. Jerison 1979) and a wealth of comparative studies has attempted to determine the factors that drove brain-size evolution (Table 1). These factors generally fit into two categories—ecological and social—with different studies supporting the importance of one category over the other. Pioneering work by Clutton-Brock and Harvey (1980) pointed to the role of ecology, with factors such as diet and home-range size explaining a proportion of interspecific variation in relative brain size. Ecological hypotheses posit that large brains with presumed greater cognitive ability (e.g. Deaner et al. 2007) are adaptations to deal with ecological problems, such as remembering the location of fruiting plants (see also Harvey et al. 1980). Later studies focused on the role of sociality in promoting larger brains and reported relationships between relative brain size and different metrics of social behavior (e.g. Dunbar 1998). Sociality and ecology may also interact. The social-brain hypothesis (*sensu stricto*) emphasizes large brains as an adaptation to ecological problem solving through social interactions (Dunbar 1992, 1998; Barton 1996; Dunbar and Shultz 2017). In addition, brain-size adaptation must balance energetic constraints. Large brains are energetically expensive, in humans accounting for about 20% of resting metabolic rate in adults, ~52% in infants, and peaking at ~66% during childhood (Kuzawa et al. 2014). Large-brained species may be those that have been better able to meet or have evolved solutions to these constraints (Aiello and Wheeler 1995; Martin 1996; Wrangham et al. 1999; Isler and van Schaik 2009); for example through greater maternal investment (Martin 1996; Powell et al. 2019). Despite decades of research, however, the drivers of primate brain-size evolution remain in dispute (Table 1; see also Whiting and Barton 2003; Dunbar and Shultz 2017).

**Table 1:** Studies testing the role of ecological and/or sociality metrics as an explanation for primate brain evolution

Study	Focus	Broad hypotheses tested	Factors found to affect brain size	*N*	Statistical approach
Clutton-Brock and Harvey 1980	Brain	Ecology, sociality	Diet, home-range size	≤30 species	OLS and MA
Dunbar 1992	Neocortex	Ecology, sociality	Group size	38 Genera	RMA
Barton 1996	Neocortex	Ecology, sociality	Group size, diet	43 Species	RMA
Joffe and Dunbar 1997	Primary visual cortex	Sociality	Social group size	27 Species	RMA
Kudo and Dunbar 2001	Neocortex	Sociality	Social partners (grooming clique size), total group size	31 Species	RMA
Reader and Laland 2002	Executive brain ratio, volume, residual^a^	Sociality	Social learning, innovation, tool use	29–30 Species	RMA
Lindenfors 2005	Neocortex	Sociality	Female group size	20 Species	RMA
Schillaci 2006	Brain	Sociality	Mating system, body mass dimorphism	31 Species	RMA
Shultz and Dunbar 2007	Brain	Diet, sociality	Diet, group size, social system, stratification, diurnal vs. nocturnal	45 Species	PGLS with BM
Dunbar and Shultz 2007	Brain	Ecology, behavior, life-history	Diet, home-range size, day range size, group size	24 Species	OLS
Perez-Barberia et al. 2007	Brain	Sociality	Social Group Associations, group size	42 Species	PGLS with BM
Maclean et al. 2009	Brain	Ecology, sociality	Diet and activity pattern	19 Lemur species	RMA and Independent Contrasts
Sandel et al. 2016	Neocortex	Sociality	Group size	23 Species	PGLS with BM
DeCasien et al. 2017	Brain	Ecology, sociality	Diet	144 Species	PGLS with BM
Street et al. 2017	Brain	Sociality, life history	Longevity, social learning, social group size	186 Species	Bayesian Mixed Models
Powell et al. 2017	Brain	Ecology, sociality	Home range size, activity period, diet	99 Species	PGLS with BM
Todorov et al. 2019	Hippocampus	Ecology, sociality	Group size, home range size	43 Species	PGLS with BM
Current study	Brain	Ecology, sociality	Diet, social/mating system, group size	128 Species	PGLS with OU

OLS = ordinary least squares regression, equivalent to a model of instant adaptation; MA = major axis regression; RMA = reduced major axis regression; PGLS = phylogenetic generalized least squares; BM = Brownian-motion; OU = Ornstein–Uhlenbeck process.

Notes: Table focuses on external selective factors driving brain evolution.

^a^All metrics focused on regions that function in innovation and social learning—neocortex and striatum.

Conflicting results from comparative studies are partly attributable to a variety of methodological issues, some of which have only recently begun to be addressed. First, small sample sizes (both intra- and inter-specific) and suboptimal quality of the underlying data can lead to errors (e.g. Smith and Jungers 1997; Borries et al. 2013; Sandel et al. 2016; Gilbert and Jungers 2017). Second, misinterpretation of statistical significance or model-selection criteria as measures of biological effect can be misleading. This is particularly pertinent when the absence of statistical significance is misinterpreted as evidence for the absence of a biological effect. Third, testing factors one at a time rather than in a multivariate fashion ignores associations that can be driving results (Dunbar and Shultz 2017). Fourth, few comparative studies have accounted for observation or measurement error in species means (but see Grabowski et al. 2016), which can cause inaccurate parameter estimates (Hansen and Bartoszek 2012; Garamszegi 2014; Morrissey 2016). Finally, statistical techniques to correct for shared ancestry vary dramatically in underlying models of trait evolution, and differences in those models can lead to different conclusions and interpretations (Garamszegi and Mundry 2014; Hansen 2014).

Phylogenetic generalized least squares with Brownian-motion assumptions for the residuals (i.e. assuming phylogenetic covariances proportional to shared branch length) is currently the most widely used approach to test hypotheses about brain-size evolution (Table 1) but is not a suitable model to study adaptation to external factors (Hansen 1997, 2014; Hansen and Orzack 2005; Labra et al. 2009; Uyeda et al. 2018; Moen et al. 2022). The main problem is that this approach implicitly assumes that adaptation is instantaneous. This is not only incompatible with the simultaneous assumption of phylogenetic correlations in the residuals, but also neglects the past history of associations between species and the niches they are adapting to. A species that has only had a short association with a given selective regime is expected to be less influenced by this regime than a species with a long history of association. Furthermore, past associations may still have an influence on the current state of the trait if the species have only recently shifted to their current niches. These effects all depend on the rate of adaptation. It is essential to estimate this rate and then use it to set up a consistent model of the evolutionary process and a correct comparative analysis (Hansen 1997; Hansen et al. 2008; and see Methods below). As adaptation is at the core of most major hypotheses about brain-size evolution, the common reliance on an unsuitable evolutionary model could be a major issue in comparative studies of brain size.

Here we test hypotheses on the role of ecology and sociality in primate brain-size evolution using phylogenetic comparative methods specifically designed to jointly estimate and account for the historical effects of adaptation and shared ancestry (Hansen 1997; Hansen et al. 2008). Our analysis uses the largest current sample of wild-caught primate average brain and body sizes with observation error—183 species—complemented by newly compiled diet data from field studies and sociality data from published compilations. Our goal is to move beyond testing whether particular predictors had a significant effect, toward a quantitative understanding of how diet and sociality have affected brain-size evolution through the history of primates.

## Materials and Methods

### Brain- and Body-Size Data

Average endocranial volume and body mass from 183 primate species based on 3506 individual observations for brain size and 6608 individual observations for body mass were taken from Isler et al. (2008). For our main analyses, each species mean was represented by at least one male and one female. Measurement variance due to estimation error in species means for both log endocranial volume (in cubic centimeters or *cc*) and log body mass (in grams or *g*) was calculated from the standard errors of the means following Grabowski et al. (2016; see also Tsuboi et al. 2018).

### Diet Metrics

We used percentage feeding time on food items compiled from field studies, supplemented by published compilations of field studies (Campbell et al. 2011; Powell et al. 2017), and divided into four categories following Nunn and van Schaik (2002): 1) fauna (e.g. insects and other small animals), 2) plant reproductive parts, 3) plant vegetative parts, and 4) plant exudates including gum. Percentage feeding time in each category was converted into two broader categorical variables—frugivorous/nonfrugivorous and folivorous/nonfolivorous—based on whether plant reproductive parts (e.g. fruit, flowers, seeds, buds, nectar), or plant vegetative parts (e.g. leaves, roots, and other plant parts) occupied a larger percentage of feeding time than the three other broad categories listed above (see also Sailer et al. 1985; Powell et al. 2017), and this scheme was kept constant across species. We chose this simple categorical scheme rather than analyzing continuous percentages or more detailed classification schemes for two reasons. First, because percentage data are likely to violate the unboundedness, homoscedascity, and normality assumptions of standard comparative methods based on Brownian-motion or Ornstein–Uhlenbeck processes, and second, because classification into more dietary categories will generate more parameters relative to the available number of species (see Text S1). Following Powell et al. (2017) we also ran separate analyses in which folivores were restricted to only those species with clear physiological adaptations for folivory (Hladik 1978; Chivers and Hladik 1980; Clutton-Brock and Harvey 1980).

### Sociality Metrics

We used average group size both as a continuous variable and as a discrete variable with five categories (groups of 1–5, 6–10, 11–25, 26–50, and >50 individuals) based on data from Powell et al. (2017) and supplemented by data from DeCasien et al. (2017). We used a four-way social system (solitary, pair-living, unimale and multifemale, multimale and multifemale) and a five-way mating system (spatial polygyny, monogamy, polyandry, harem polygyny, polygynandry) following the classification of DeCasien et al. (2017), supplementing their published dataset with 32 additional species from the same published compilations (Plavcan 1999; Thorén et al. 2006; Shultz et al. 2011). Finally we reclassified social system into the metric of social complexity following DeCasien and Higham (2019) using a three-way category scheme based on general group type—solitary, pair-living, or group-living. We did not run models with group-size categories, social system, and mating system together because these factors are closely related to each other. We also did not run models with different categorizations of sociality or average group size and group-size categories at the same time for the same reason.

### Activity Period

Although not a primary focus of our study, we included activity period as a covariate because previous analyses have suggested that it has a role in brain evolution (Barton 1996, 1999; Barton et al. 1999; Powell et al. 2017; DeCasien et al. 2019). Based on data from Powell et al. (2017) and supplemented by data from published compilations (MacLean et al. 2009; Shultz et al. 2011; Mittermeier and Wilson 2013; Santini et al. 2015), we treated activity period as a dichotomous variable (diurnal/nocturnal). Following Powell et al. (2017) a few cathemeral species were classified as diurnal. While activity period is generally grouped with ecological variables such as diet (e.g. Barton 1996), it has been shown to interact with sociality metrics (Shultz et al. 2011). It is thus not clear to which group it should be assigned.

Note that we specifically focus on factors that underlie the role of ecology and sociality; hence behavioral factors previously identified as influencing brain size, such as cognitive performance (e.g. MacLean et al. 2014) are not included here, though they are undoubtedly important.

### Phylogeny

We used the dated consensus tree from version 3 of 10Ktrees website using the Genbank taxonomy (Arnold et al. 2010), as has been done in numerous recent studies on brain-size evolution in primates (DeCasien et al. 2017; DeCasien and Higham 2019; Todorov et al. 2019). Our individual analyses were based on subsets of species due to missing data for some predictor variables. The main set of analyses was based on 128 species with complete diet, sociality, and activity-period data; our univariate analyses were subsets of the 183 species dataset. We note that while there are by some accounts more than 500 living species of primates (Estrada et al. 2018), the phylogeny used here is based on 301 species. Our complete data on endocranial volume, body mass, diet, sociality, and activity period are provided in the supplementary material.

### Comparative Approach

Following Hansen (1997) we model evolution of log endocranial volume as an Ornstein–Uhlenbeck process around a (primary) optimal state that is a function of different predictor variables (diet, social system, etc.) mapped as regimes on the phylogeny for the categorical predictors and of a randomly changing predictor variable for log group size (Hansen et al. 2008). Following Grabowski et al. (2016) we augment this with a “direct” effect of log body mass such that evolutionary changes in body size are associated with an immediate correlated response in brain size. The model is described by the stochastic differential equations:


$$dy=-\alpha (y-\theta (z,g))dt+bdx+\sigma_{y}{dB}_{y}$$

$$dg=\sigma_{g}{dB}_{g}$$

$$dx=s_{x}{dB}_{x}$$

where *y* is log endocranial volume, *g* is log group size, and *x* is log body mass. The *dB*_*y*_*dB*_*g*_ and *dB*_*x*_ are independent white-noise processes multiplied by their standard deviations *σ*_*y*_, *σ*_*g*_, and *σ*_*x*_. The α-parameter quantifies the rate of adaptation toward a niche-specific optimum, *θ*, modeled as a function of ecological predictors, *z*, such as diet and social system, mapped on the phylogeny, as well as *g*, log group size, modeled as evolving according to a Brownian-motion process. The α-parameter can be expressed as a phylogenetic half-life, *t*_1/2_ = ln(2)/*α*, the time it takes for the response variable to evolve in expectation half the distance from its ancestral state toward the current optimum. The parameter *b* gives the direct effect of changes in body size on brain size, as may be expected from a within-species, static, allometry. In the results, we report σ_*y*_ by reparameterizing as the stationary variance of the Ornstein–Uhlenbeck process as $R^{2}$ the expected residual variance when species have evolved under constant adaptive regimes for a long period of time.

In many of our analyses the phylogenetic half-life becomes very large at the same time as the optima are estimated to be far outside the range of the species data. This is not uncommon in Ornstein–Uhlenbeck models of adaptation but requires a reparameterization and some reinterpretation (Hansen 1997). An infinite half-life is a characteristic of a Brownian motion, but when this happens simultaneously with infinitely large optima, the deterministic (adaptation) part of the model does not vanish, and the model converges on a Brownian motion with niche-specific deterministic trends. These trends can be reliably estimated as  Δ  even when the *α* and *θ* parameters are individually inaccurate (Hansen 1997). The biological interpretation is that the species are far from their niche-specific optima and continuously evolving toward them at niche-specific rates. We estimate these “adaptive” trends, *τ*(*z*, *g*) with units of trait change per tree height, as functions of ecological variables such as diet and social systems in the same way as for the primary optima described above. Note that this trend model is different from standard Brownian-motion-based PGLS in that the deterministic prediction for each species is a weighted sum of the different trends it has experienced through its history. It thus incorporates (or models) historical information about past selective regimes.

Since all species are extant, the trends are only meaningful as contrasts with each other, and not as estimates of absolute trends (Hansen 1997). As our phylogeny is scaled to unit height = 1 (original height 73 million years or myr), a contrast of 50% per tree height when switching from spatial polygyny to polygynandry would mean that relative endocranial volume is expected to be 50% larger in the polygynandric regime than it would have been in the spatial polygynic regime for species that have remained in those regimes over their entire history from the root of the tree.

We start by using *bayou* version 2.2.0 (Uyeda and Harmon 2014) to search for shifts in the adaptive optima of the Ornstein–Uhlenbeck process and allometric parameters across the phylogeny without an *a priori* hypothesis. Here we use the latest version of *bayou* to detect shifts in allometric exponents for the relationship between log endocranial volume and log body mass (see also Miller et al. 2019), a function that was not available prior to version 2.0. We ran six chains, two for each model, each for three million generations. Convergence was measured using Gelman’s R statistic and was less than 1.005 for *α*, 2.63[0.43−∞] and the log likelihood. Following Uyeda et al. (2017), we initialized the MCMC without any shift proposals for the first 10,000 generations to improve the fit of the model. Priors for the parameters as defined above were set to:


α∼logN(μ=log(0.5),σ=2.0)

$$\sigma_{y}^{2}∼\mathrm{half-Cauchy}(\mu =0,\sigma =0.1)$$

b∼N(μ=0.6,σ=0.1)

Here, the prior on *α* is a distribution of *t*_*1/2*_ in which the lower 10% is equivalent to 1 million years or less, and the upper 10% starts at two times the height of the tree. The prior on *b* is based on the global slope estimate for primates found in Grabowski et al. (2016). Model comparisons were performed using Bayes factors following Uyeda et al. (2017).

We next tested for relationships between relative brain size and metrics of diet and sociality with *slouch* 2.1.4 (Hansen et al. 2008; Kopperud et al. 2020), using the location of the *bayou*-discovered regime shifts to inform our analyses. *Slouch* uses generalized least squares to estimate primary optima or trends in the Ornstein–Uhlenbeck framework conditionally on the other parameters of the model, which are estimated by maximum likelihood. Contrasts between trends with standard errors are calculated in *slouch*, and we give a specific example of how this is done in Text S4.

Apart from some colinear combinations indicated above, we compared all possible combinations of factors hypothesized to influence brain size with likelihood using AICc. All models accounted for measurement error in both log endocranial volume and log body mass following previous approaches (Hansen and Bartoszek 2012; Grabowski et al. 2016), a feature that is built into *slouch* (Kopperud et al. 2020). We assessed the fit of the model for the primary optimum (i.e. the adaptive landscape) with a residual *R*^*2*^; that is, the percent of residual variance explained by the predictors after accounting for body mass. We did this because log body mass itself explains a large proportion of the variance in log endocranial volume—around 80% on average. Thus, explaining half of the remaining 20%, though only 10% of the total variance, is arguably a substantial result, which is made clear by this metric.

To assess uncertainty in the evolutionary history of the discrete predictors: diet, social categories, and activity period were first mapped on the full phylogeny using stochastic character mapping using the make.simmap function from phytools (Revell 2011) to fit a continuous time-reversible Markov-chain model, which was then used to simulate character states on the phylogeny. For each model including discrete predictors, we used AICc to choose the structure of the transition rate matrix from the possibilities of equal rates, symmetric rates, or all rates different (Table S1). After running all simulated mappings in *slouch*, their AICc distributions were used to qualitatively inform model selection. The final regime assignments were the maximum a posteriori regime estimates for each node in the Markov-chain model (Figs. S1-S8) with the transition rate matrix with the lowest AICc score. None of the results were qualitatively changed by the analyses of stochastic maps and we do not discuss these further (see Text S2).

## Results

### Shifts in Optima

**Figure 1.**
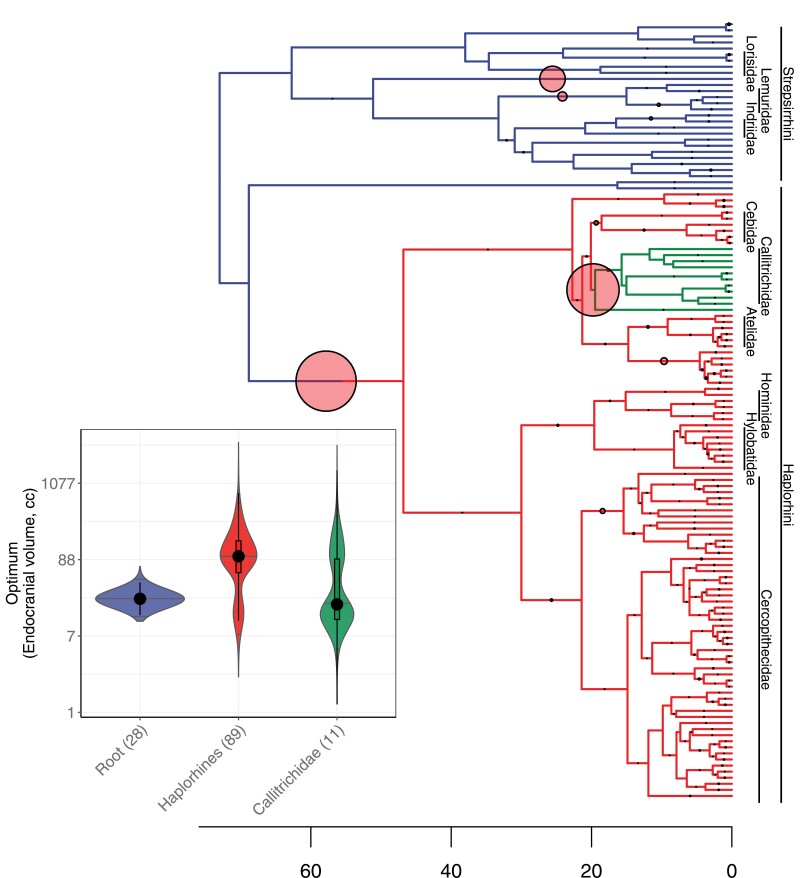
Time-calibrated primate phylogeny showing regimes for best fitting bayou model of log ECV (cc) on log Body Mass (g). Regime shifts are shifts in the intercept of the allometric equation with a posterior probability greater than 0.5. Diameter of circles is proportional to the posterior probability of a shift. The tree height is 73 myr. Inset shows estimated optima for the two regime shifts with the black dot and the box indicating the median and the interquartile range of the posterior distribution. Number of species in that clade is shown in parenthesis.

The *bayou* analysis favored two shifts in the relative brain-size optimum with posterior probabilities (*pp*) higher than 0.5. One near the origin of the haplorhines after the split from tarsiers and the other within platyrrhines (*pp* = 0.90, and *pp* = 0.78, respectively; Fig. 1; Fig. S9; Table S2). Therefore, we used the two *bayou*-located shifts to inform our *slouch* analyses. There was no evidence for changes in the allometric slope, 0.56 (95% highest posterior density (HPD) of 0.5–0.62), and the shifts in the intercept model were supported with a Bayes Factor of 11.04 over a model with no change and 9.67 over a model with shifts in both intercept and slope. The posterior median phylogenetic half-life for this model was *t*_*1/2*_ = 67% of tree height with a 95% HPD of 32%-∞ (Table S2; Text S3). A third possible shift, along the lineage leading to the Aye-aye *Daubentonia madagascariensis*, had a posterior probability slightly below our 0.5 cutoff (*pp* = 0.389). As pointed out by Uyeda et al. (2017), it is not possible to determine if shifts on branches leading to singleton taxa are the result of shifts in the slope or in the intercepts of allometric relationships.

### Adaptation to Diet and Social Parameters

**Figure 2.**
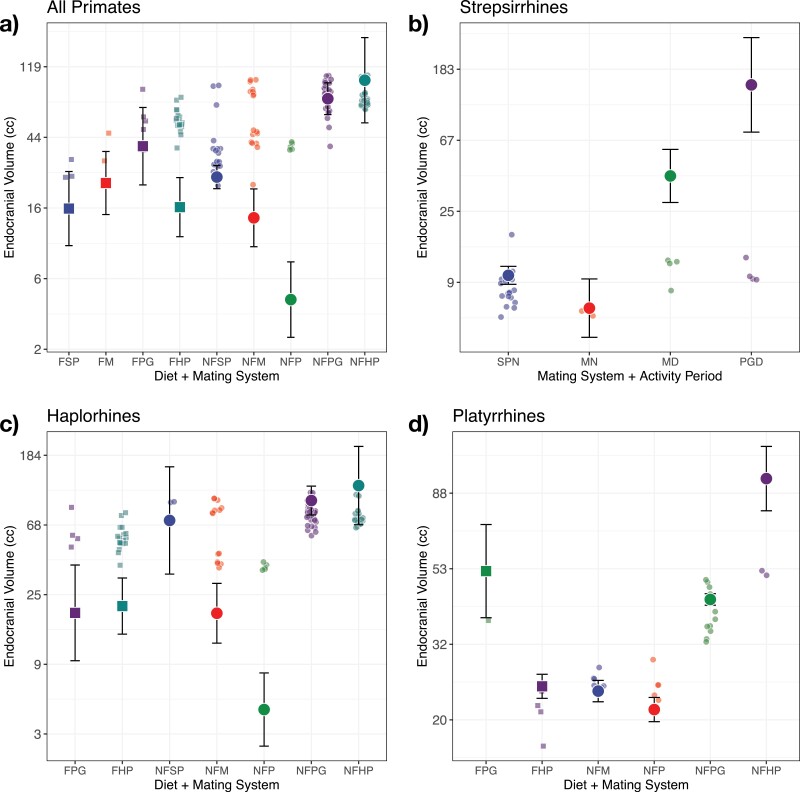
Brain size as a function of ecological factors. Mean brain size of each species is plotted as a function of its current ecological or social factors for a) all primates. b) Strepsirrhines plus tarsiers. c) Haplorhines minus tarsiers. d) Platyrrhines. In a), b), and c) we show the predicted brain sizes from the best-fitting evolutionary trend model if the species had evolved in the same regime from the root of the phylogeny assuming for illustration that they all started from the global mean brain and body mass. For platyrrhines the best model was an Ornstein–Uhlenbeck model with fixed optima and not a trend model. Hence, in d) we show the estimated primary optima for the regimes. In all cases the evolutionary model included body size and the plotted species mean brain sizes are residuals from the allometric model. Note that the species values are often systematically different from the evolutionary predictions. This is because the species may not have evolved in their current selective regime for a long time, and thus display a lag in adaptation. The error bars are standard errors, conditional on the maximum likelihood estimates for *α* and N. Abbreviations are Folivorous Spatial Polygyny (FSP), Folivorous Monogamy (FM), Folivorous Polygynandry (FPG), Folivorous Harem Polygyny (FHP), Non-folivorous Spatial Polygyny (NFSP), Non-folivorous Monogamy (NFM), Non-folivorous Polygyny, (NFP), Non-folivorous Polygynandry (NFPG), Non-folivorous Harem Polygyny (NFHP) in a), b), and c) with subsets in b) along with a nocturnal (N) or diurnal (D) activity period. Folivores are squares, nonfolivores are circles.

The best model for the evolution of brain size across primates as a whole included distinct regimes for diet (strict folivorous/nonfolivorous) and mating system, with group size added as a continuous variable. Together these factors explained 31% of the residual variance in brain size after accounting for body size (Table 2; Tables S3, S4). The phylogenetic half-life for this model was 263% percent of the tree height (2-unit support interval 43% of tree height-∞), which means that relative brain-size evolution behaves roughly as Brownian motion with distinct evolutionary trends in the different selective regimes (Fig. 2a).

**Table 2:** *Slouch* results for all primates showing the best model, its parameter estimates and type, and the allometric model (i.e. only log body mass as a predictor)^a,b^

Group	*N*	Predictors	Parameters	Regime trends	Direct effect	Evolutionary	$t_{1/2}$	$V_{y}$	$R^{2}$ (%)	Res. $R^{2}$(%)	Δ AICc
				Intercepts	Slope	Slope					
All primates	128	Diet (strict folivorous/ nonfolivorous) + group size + mating system + body mass	Folivorous harem polygyny	–2.87±1.72			2.63[0.43−∞]	0.18[0.04−0.29]	87.0	34.9	0.0
				
			Folivorous monogamy	–1.70±1.79							
			Folivorous polygynandry	0.41±2.19							
			Folivorous spatial polygyny	–3.17±2.05							
			Nonfolivorous harem polygyny	–4.19±2.37							
			Nonfolivorous monogamy	–3.29±1.55							
			Nonfolivorous polyandry	–7.77±1.99							
			Nonfolivorous polygynandry	3.25±1.01							
			Nonfolivorous spatial polygyny	–1.32±0.20							
			Log body mass		0.56±0.02						
			Log average group size			–0.04±0.02					
All primates	128	Body mass	Intercept	–1.30±0.24			∞[0.86−∞]	0.35[0.12−0.45]	80.0	0.0	21.5
			Log body mass		0.59±0.03						

Notes: In each case, log endocranial volume is the response variable and log body mass is included as a direct effect predictor.^a^Results include sample size (*N*), estimated parameters, the phylogenetic half-life ($t_{1/2}$) in units of tree height with 2-unit support interval shown in brackets, the stationary variance ($V_{y}$) in units of squared trait units (log ECV in cc) per unit tree height with 2-unit support interval shown in brackets, the variance explained by the model ($R^{2}$) shown in %, the extra variance explained by the model after accounting for the variance explained by body mass (Res. $R^{2}$), and the difference between the AICc score of the best model and the Brownian-motion model with body mass as the sole predictor (ΔAICc).

^b^Estimated parameters include Regime Optima or Trends for the effects of the categorical predictors – the former from an Ornstein-Uhlenbeck model [in trait units (log ECV in cc)], the latter from an Ornstein-Uhlenbeck model as half-life goes to infinity [in units of trait units (log ECV in cc) per tree height], the Direct Effect Slope for the relationship between brain and body mass [in units of trait units (log ECV in cc) per change in log body mass], and the Evolutionary Slope for the relationship between brain mass and group size [in units of trait units (log eCV in cc) per change in log Group Size].

Based on reconstructed shifts in diet and mating systems (Fig. S10), one of the largest upward shifts in trends occurred with a change from spatial polygyny to polygynandry in species maintaining a nonfolivorous diet (Fig. 2a). The estimated contrast for this shift was 1.11 ± 0.27 log *cc* per tree height, corresponding to an Exp[1.11] = 303% difference or a threefold change over the time span of the phylogeny (Table S4; Text S4). This coincides with one of the shifts located by *bayou* (Fig. 1), which occurred after tarsiers split off from other haplorhines. The other *bayou*-located shift, near the origin of the Callitrichidae, is the largest downward shift with a contrast of −2.85 ± 0.52 log *cc* per tree height, corresponding to a 94% downward shift in the trend associated with a change from polygynandry to polyandry while maintaining a nonfolivorous diet, but we caution that this latter shift is based on a small number of polyandrous species.

The largest predicted shifts were for species switching to nonfolivorous harem polygyny from any of the other regimes, which occurred multiple times across the phylogeny. The second largest shift is associated with switching to nonfolivorous polygynandry (Fig. 2a and Table 2; Fig. S10).

Group size had a small negative effect on relative brain size with an evolutionary slope of −0.08 ± 0.03 log *cc* per tree height, meaning that a doubling of group size would lead to a 2.7% decrease in brain size over the time span of the phylogeny (Text S4).

Because shifts in diet happen in the context of a variety of mating systems, it is difficult to fully disentangle the two, but it is clear that a folivorous diet is associated with a downward shift in the brain-size trend (Fig. 2a; Fig. S10). Shifting from nonfolivorous polygynandry to the folivorous variant is predicted to decrease the adaptive trend with a contrast of −0.67 ± 0.54 log *cc* per tree height—an almost 50% decrease over the time span of the phylogeny. Similarly, shifting from nonfolivorous to folivorous spatial polygyny decreases the trend with −0.44 ± 0.53 log *cc* per tree height—and 36% reduction over the time span of the phylogeny (Fig. 2a; Table S4).

A model with only the two *bayou*-located regime shifts alone or combined with group size did not improve the fit (Table S5). This shows that while some shifts may correspond to those located by *bayou*, the combination of diet categories and sociality has better explanatory power.

Using male or female averages separately (Text S7) or rerunning analyses with single predictors (Text S8) gave results consistent with the main findings both for primates as a whole and for the subclades. The smaller residual *R*^*2*^ values for single predictors illustrate the importance of using multiple predictors in explaining relative brain size (Text S8).

### Variation within Taxonomic Groups

Based on the *bayou*-located shifts, we divided the phylogeny into three major subsets—strepsirrhines plus tarsiers, haplorhines minus tarsiers, and platyrrhines. We used these subsets to determine if relative brain size evolved differently in the different groups. As only one haplorhine species included here was nocturnal (*Aotus trivirgatus*), activity period was not included as a predictor in the haplorhine or platyrrhine subsets.

#### Strepsirrhines.

—For strepsirrhines plus tarsiers (*n* = 28), the best model included group size, mating system, and activity period, together explaining 65% of the residual variance and 95% of the total variance in brain size (Table 3; Tables S6, S7). The phylogenetic half-life for this model was infinity with a 2-unit support interval of 43%–∞, which means that evolution behaves as a Brownian motion with distinct trends in the different selective regimes (Fig. 2b). Based on reconstructed shifts (Fig. S11), we found large changes associated with two regime switches. Going from nocturnal monogamy to diurnal polygynandry was associated with a contrast of 3.15 ± 0.71 log *cc* per tree height, which would correspond to a 23-fold difference in brain size over the time span of the phylogeny. Switching from nocturnal to diurnal monogamy was associated with a contrast of 1.86 ± 0.46 log cc per tree height, which would correspond to a 6-fold difference over the time span of the phylogeny. These extreme differences are of course not realized, as most species have spent relatively short amounts of time in the diurnal monogamous and polygynandric regimes (Fig. S11), and are thus predicted to be far from their optimal brain sizes (Fig. 2b). Within mating-system and activity-period categories, group size had a negative effect, with an evolutionary slope of −0.53 ± 0.11 log *cc*, meaning that a doubling of group size would lead to a 17% reduction over the time span of the phylogeny (Text S5). Because this negative effect of group size did not appear in the other subclades, it is likely that this result in strepsirrhines drives the effect found for all primates.

**Table 3:** *Slouch* results for strepsirhines showing the best model, its parameter estimates and type, and the allometric model (i.e. only log body mass as a predictor)^a,b,c^

Group	*N*	Predictors	Parameters	Regime trends	Direct effect	Evolutionary	$t_{1/2}$	$V_{y}$	$R^{2}$ (%)	Res. $R^{2}$(%)	Δ AICc
				Intercepts	Slope	Slope					
Strepsirrhines	28	Group size + mating system + activity period + body mass	Monogamy diurnal	0.14±0.49			∞[0.43−∞]	0.1[0.02−0.22]	94.5	64.5	0.0
			Monogamy nocturnal	–1.72±0.48							
			Polygynandry diurnal	1.42±0.77							
			Spatial polygyny nocturnal	–1.26±0.23							
			Log body mass		–0.56±0.03						
			Log average group size			-0.53±0.11					
Strepsirrhines	28	Body mass	Intercept	–1.47±0.35			∞[0.18−∞]	0.06[0.04−0.43]	84.5	0.0	13.6
			Log body mass		0.59±0.05						

Notes: In each case, log endocranial volume is the response variable and log body mass is included as a direct effect predictor.^a^Results include sample size (N), estimated parameters, the phylogenetic half-life ($t_{1/2}$) in units of tree height with 2-unit support interval shown in brackets, the stationary variance ($V_{y}$) in units of squared trait units (log ECV in cc) per unit tree height with 2-unit support interval shown in brackets, the variance explained by the model ($R^{2}$) shown in %, the extra variance explained by the model after accounting for the variance explained by body mass (Res. $R^{2}$), and the difference between the AICc score of the best model and the Brownian-motion model with body mass as the sole predictor (ΔAICc).

^b^Estimated parameters include Regime Optima or Trends for the effects of the categorical predictors – the former from an Ornstein-Uhlenbeck model [in trait units (log ECV in cc)], the latter from an Ornstein-Uhlenbeck model as half-life goes to infinity [in units of trait units (log ECV in cc) per tree height], the Direct Effect Slope for the relationship between brain and body mass [in units of trait units (log ECV in cc) per change in log body mass], and the Evolutionary Slope for the relationship between brain mass and group size [in units of trait units (log eCV in cc) per change in log Group Size].

^c^Plus tarsiers.

#### Haplorhines.

—For haplorhines excluding tarsiers (*n* = 100), the best model included diet categories (strict folivorous/nonfolivorous) and mating system explaining 32% of the residual variance (Table 4; Tables S8, S9). As the shift to polygynandry from spatial polygyny had already taken place at the root of the haplorhine clade, it is likely that diet and mating system may be playing a further role in brain-size patterning within the haplorhines. The phylogenetic half-life for this model was also infinity (2-unit support interval 43%–∞). Overall trends (Fig. 2c; Fig. S12) were similar to the results for all primates though effects associated with nonfolivorous spatial polygyny had higher standard error due to the small number of species in this regime. In addition, species displaying folivorous polygynandry and folivorous harem polygyny were distant from their predicted brain sizes (Fig. 2c) as expected from the short amount of time they have spent in these regimes (Fig. S12). The model predicts that brain size is still decreasing in these species.

**Table 4:** *Slouch* results for haplorines showing the best model, its parameter estimates and type, and the allometric model (i.e. only log body mass as a predictor)^a,b,c^

Group	*N*	Predictors	Parameters	Regime trends	Direct effect	$t_{1/2}$	$V_{y}$	$R^{2}$ (%)	Res. $R^{2}$(%)	Δ AICc
				Intercepts	Slope					
Haplorhines	100	Diet (strict folivorous/ nonfolivorous) + mating system + body mass	Folivorous harem polygyny	–6.77±1.75		∞[0.43−∞]	0.2[0.04−0.29]	84.2	32.2	0.0
			Folivorous polygynandry	–7.17±3.00						
			Nonfolivorous harem polygyny	0.86±2.37						
			Nonfolivorous monogamy	–7.10±1.80						
			Nonfolivorous polyandry	–13.28±2.18						
			Nonfolivorous polygynandry	–0.02±0.29						
			Nonfolivorous spatial polygyny	–1.21±3.43						
			Log body mass		0.52±0.03					
Haplorhines	100	Body mass	Intercept	–0.74±0.31		∞[0.67−∞]	0.33[0.08−0.43]	76.6	0.0	22.9
			Log body mass		0.58±0.03					

Notes: In each case, log endocranial volume is the response variable and log body mass is included as a direct effect predictor.^a^Results include sample size (N), estimated parameters, the phylogenetic half-life ($t_{1/2}$) in units of tree height with 2-unit support interval shown in brackets, the stationary variance ($V_{y}$)in units of squared trait units (log ECV in cc) per unit tree height with 2-unit support interval shown in brackets, the variance explained by the model ($R^{2}$) shown in %, the extra variance explained by the model after accounting for the variance explained by body mass (Res. $R^{2}$), and the difference between the AICc score of the best model and the Brownian-motion model with body mass as the sole predictor (ΔAICc).^b^Estimated parameters include Regime Optima or Trends for the effects of the categorical predictors – the former from an Ornstein-Uhlenbeck model [in trait units (log ECV in cc)], the latter from an Ornstein-Uhlenbeck model as half-life goes to infinity [in units of trait units (log ECV in cc) per tree height], the Direct Effect Slope for the relationship between brain and body mass [in units of trait units (log ECV in cc) per change in log body mass].

^c^Minus tarsiers.

#### Platyrrhines.

—For platyrrhines (*n* = 32), diet categories (strict folivorous/nonfolivorous) and mating system explained 93% of the residual variance, and together with body mass, 98% of the total variance in log brain size (Table 5; Tables S10, S11). In contrast to the other clades, the phylogenetic half-life was much shorter, 6% (2-unit support interval 6–92%) of the total height of the platyrrhine phylogeny (22 myr) corresponding to 1.32 myr. This suggests that brain size is evolving around estimable evolutionary optima influenced by the combination of diet and mating system (Fig. 2d; Fig. S13). Species displaying folivorous harem polygyny, nonfolivorous monogamy, nonfolivorous polyandry, and nonfolivorous polygynandry are all near their adaptive optima, though shifting from the latter to any of the former decreases the optimal brain size in platyrrhines by 52% (Table 5; Text S6). Alternatively, shifting to nonfolivorous harem polygyny from nonfolivorous polygynandry increases the brain-size optima by 223%, though species in the former regime are distant from their predicted brain-size optima (Fig. 2d) as expected from the short amount of they have spent in these regimes (Fig. S13; Text S6).

**Table 5:** *Slouch* results for platyrrhines showing the best model, its parameter estimates and type, and the allometric model (i.e. only log body mass as a predictor)^a,b^

Group	*N*	Predictors	Parameters	Regime trends	Direct effect	$t_{1/2}$	$V_{y}$	$R^{2}$ (%)	Res. $R^{2}$(%)	Δ AICc
				Intercepts	Slope					
Platyrrhines	32	Diet (strict folivorous/ nonfolivorous) + mating system + body mass	Folivorous harem polygyny	–1.53±0.30		0.06[0.06−0.92]	0.01[0.01−0.05]	98.3	92.5	0.0
			Folivorous polygynandry	–0.79±0.45						
			Nonfolivorous harem polygyny	–0.15±0.35						
			Nonfolivorous monogamy	–1.57±0.21						
			Nonfolivorous polyandry	–1.66±0.21						
			Nonfolivorous polygynandry	–0.95±0.27						
			Log body mass		0.62±0.03					
Platyrrhines	32	Body mass	Intercept	–1.84±0.51		∞[0.18−∞]	0.53[0.06−0.96]	77.6	0.0	18.9
			Log body mass		0.71±0.07					

Notes: In each case, log endocranial volume is the response variable and log body mass is included as a direct effect predictor.

^a^Results include sample size (N), estimated parameters, the phylogenetic half-life ($t_{1/2}$) in units of tree height with 2-unit support interval shown in brackets, the stationary variance ($V_{y}$) in units of squared trait units (log ECV in cc) per unit tree height with 2-unit support interval shown in brackets, the variance explained by the model ($R^{2}$) shown in %, the extra variance explained by the model after accounting for the variance explained by body mass (Res. $R^{2}$), and the difference between the AICc score of the best model and the Brownian-motion model with body mass as the sole predictor (ΔAICc).^b^Estimated parameters include Regime Optima or Trends for the effects of the categorical predictors – the former from an Ornstein-Uhlenbeck model [in trait units (log ECV in cc)], the latter from an Ornstein-Uhlenbeck model as half-life goes to infinity [in units of trait units (log ECV in cc) per tree height], the Direct Effect Slope for the relationship between brain and body mass [in units of trait units (log ECV in cc) per change in log body mass].

## Discussion

Overall, we found a relative decrease in brain size when shifting from frugivory to folivory, and a relative increase when shifting to more complex social systems. As the predictors of brain size for primates in general appear to be generated by a combination of differences between and within suborders, we discuss each clade in turn rather than attempting to interpret our detailed results as being general to all primates. This being said, our finding that both diet and sociality effect brain-size evolution stands in contrast to a number of recent analyses that favored ecological rather than social factors (e.g. DeCasien et al. 2017; Street et al. 2017), or social rather than ecological factors (e.g. Todorov et al. 2019). The phylogenetic heterogeneity of these factors makes it likely that at least some of the disparate results may be an outcome of differences in taxonomic sampling. Our results also illustrate how choice of models for phylogenetic comparative analysis can affect the outcome.

### Comparisons with Previous Studies and Biological Interpretations

#### Strepsirrhines.

—Our finding that group size, mating system, and activity period affected brain-size evolution, both complement and contrast those of previous studies. In a study of 19 different lemur species, MacLean et al. (2009) concluded that diet and activity period (cathemeral, diurnal, or nocturnal), rather than social group size or pair bonding, were associated with relatively larger brains. Like us, they found a tendency for a negative effect of group size on brain size but discounted the result due to a lack of statistical power. The negative effect of group size in our study was also relatively weak. While a negative effect of group size may not support the social-brain hypothesis, it must be noted that this is a residual effect from a model with stronger effects of social system. For example, brain evolution was accelerated by a shift to diurnal polygynandry from diurnal monogamy, with the former arguably a more complex social system than the latter. We also note that the negative group-size effect in primates as a whole appears to be solely due to the negative relationship within the strepsirrhines.

MacLean et al. (2009) also reported an effect of diet on brain size based on reduced major axis regression, but no effect when using independent contrasts. This latter finding is mirrored in our results because the relationship between diet and brain size in strepsirrhines disappears if we assume strong phylogenetic signal (i.e. Brownian motion). Brain size is adapting to diet on a short time scale relative to the time span of the phylogeny, however, taking on average 4.4 myr to evolve half the distance to the new optima. This model indicates that folivory is associated with a 28% reduction in optimal brain size (Text S5).

#### Haplorhines.

Overall, we found that harem polygyny combined with a nonfolivorous diet is associated with the fastest increases in relative brain size. This result differs from other findings of a relationship between brain size and mating system in haplorhines, which pointed to monogamous (Schillaci 2006), or polygynandrous (Shultz and Dunbar 2007) species as possessing the largest brains. One explanation for these divergent results is that when mating system is considered independently from diet, the pattern is reversed, with shifting to harem polygyny from polygynandry resulting in a 48% decrease in the rate of relative brain-size evolution over the time span of the phylogeny (Text S9). Both harem polygyny and polygynandry are complex mating systems when compared with spatial polygyny or monogamy, lending support to Dunbar’s (1998) hypothesis that the ability to manage social interactions within these systems required larger brains.

On the other hand, haplorhines adapted to eating foliage appear to have relatively smaller brains. As frugivory was likely the ancestral diet for haplorhines (Supplementary Fig. S10 and see Soligo and Martin 2006), haplorhines that evolved away from this diet may have done so recently. Together with long phylogenetic half-lives, this finding suggests that brain size in folivores may continue to diverge downward relative to nonfolivores. Contrary to suggestions by Clutton-Brock and Harvey (1980) and DeCasien et al. (2017) that brain sizes tend to increase in frugivorous species, our results are more consistent with a decrease in folivorous species. As discussed further below, this does not rule out a role for frugivory in the origination or maintenance of large brains in some lineages.

Our findings illustrate how studying adaptation using methods that account for the evolutionary history of the selective environment, such as nonfolivory versus folivory, can affect the results and their biological interpretation. Our findings are consistent with suggestions that the smaller brains of folivores are the result of folivores having evolved a large stomach and digestive system (Hladik 1967), which may require shifts in energy allocation away from the brain (Aiello and Wheeler 1995; Isler and Van Schaik 2009; Fonseca-Azevedo and Herculano-Houzel 2012). An alternative interpretation is that large brains in primates compared with other mammals are maintained as an adaptation to ancestral frugivory and the associated ecological problem solving. If so, this could be an example of “stasis is data” (Gould and Eldredge 1977), where energetically expensive large brains are maintained by stabilizing selection in frugivorous lineages while lineages that no longer required these functions shifted resources to digestion.

#### Platyrrhines.

The rapid adaptation of brain size given the best-fitting model (strict diet categories + mating system) reveals the distinctive nature of platyrrhines compared with other primates. The fact that diet categories, mating system, and body mass together explain 98% of brain-size variance in platyrrhines may reflect both their narrow ecological range and their relatively recent adaptive radiation—the last common ancestor of all living platyrrhines is dated to the early Miocene, about 20 Ma (Fernandez-Duque et al. 2012), and there may not have been enough time for species to adapt to divergent selection pressures since they arose. This is illustrated by the brain sizes of species exhibiting nonfolivorous harem polygynandry being much smaller than their predicted primary optimum—given time, we predict that species in this regime will approach their optima. Our findings are complementary to Aristide et al. (2016), who suggested that brain shape, rather than size as studied here, is related to diet, locomotion, and social group size. In fact, we found that group size alone was the third best supported model, after a model including diet categories, mating system, and group size. Platyrrhines are noted for having a wide range of social systems and variation in group size and composition, but a narrow ecological range (Fernandez-Duque et al. 2012).

Our ancestral-state reconstruction (Fig. S13) indicates that nonfolivorous polygynandry was ancestral for platyrrhines and maintained in other platyrrhine lineages while the family Callitrichidae shifted to monogamy and polygynandry. The Callitrichidae appear to follow the same brain-body relationship found across strepsirrhines and tarsiers (Fig. S13). Martin (1992) referred to the Callitrichidae as a dwarfed lineage, and it is possible that the brain-body relationship is constrained to evolve in a narrow range, with Callitrichidae mirroring strepsirrhines and tarsiers through convergent evolution. This result is further supported by the *Alouatta* clade, which appears to have experienced a similar reduction in brain size in response to a shift to folivory (Fig. S13). Brain-size variation may then reflect variation in the evolutionary allometric intercept, as found in other group of vertebrates (Tsuboi et al. 2018) as well as in allometric relationships of other morphological traits (Voje et al. 2014).

### Caveats and Conclusions

Brains are multifunctional organs interacting with all aspects of an animal’s life. Consequently, we expect multiple sources of selection and constraint to have potential impact on brain size and structure. However, only a small set of potentially relevant factors have been investigated and interpreted, often in isolation from each other. While past studies have sometimes suggested a lack of relationship with this or that factor, many suffer from the issues mentioned in the introduction, including small sample sizes, misinterpretation of statistical significance as measures of biological effect, and using statistical models that do not match the biological context and question. Most likely, brain evolution has been influenced by a multitude of ecological, social, behavioral, physiological, and sensory factors that may have varied in their impact through the history of the species. The multitude of influences may be reflected in our finding of a Brownian-motion-like pattern in the residual deviations from our main models. Such a pattern is expected from many quasi-independent factors shifting constraints and local optima stochastically around the main trends. Hypothetically, evolutionary lags and phylogenetic correlations would diminish if more factors were included in the main model (see Labra et al. 2009).

A particular shortcoming for a comparative study of brain-size evolution is that we have little quantitative information on the behavioral, cognitive, and sensory features that mediate selection on brain size. As just one example, vocalizations are essential in the life of most primates and their production and interpretation require complex neuronal processing. Vocalizations are affected by body size (Hauser 1993) and reflect and influence a variety of social and ecological factors (Fitch and Hauser 1995). It is thus to be expected that shifts in these factors may alter selection on vocalizations with associated, but unfortunately unknown, effects on brain evolution.

The brain is a complex structure with many parts, and overall brain size is a crude measure of its capabilities. The size of individual parts will covary with overall brain size (Clark et al. 2001; Finlay et al. 2001; Falk 2007; but see Barton and Harvey 2000), and the hope is that a study of overall size may capture some similar insights as would have been captured by studies of individual parts. In a recent study, DeCasien and Higham (2019) tested how 33 different brain regions correlated with ecological and sociality traits, noting that past studies focused on only a limited number of regions (e.g. neocortex size) and that findings were inconsistent (e.g. Dunbar 1998; Schillaci 2008). Their results suggested that both dietary and social complexity were factors influencing the size of individual brain regions, which differed among primate groups. Such studies reveal more detail about brain evolution, but also have some shortcomings relative to studies of the whole brain in that they tend to be based on smaller sample sizes (between 17 and 58 species depending on the region analyzed in the DeCasien and Higham study), and the chosen phylogenetic comparative methods are problematic in our view. As we have illustrated, different results may appear from the assumption of different evolutionary models. It would be instructive to also base comparative analyses of brain parts on methods consistent with adaptive evolution.

There is debate as to whether cognitive abilities are best captured by absolute or relative brain size (Striedter 2005; Deaner et al. 2007; MacLean et al. 2014). In our study we used absolute brain size as a response variable, but we included a direct (allometric) effect of body size on its evolution. While we estimated the allometric effect of body size jointly with the adaptive effects, and indeed found that it explained the majority of the variance in brain size, our model of adaptation to ecological and dietary factors then apply to deviations from brain-body allometry, and in this sense to relative brain size. It is of course possible that absolute brain size and its cognitive correlates may also adapt by inducing selection on body size, or evolve as a side effect of changes in body size driven by other means (e.g. Lande 1979).

Overall, our results contradict the idea that there is one general “rule” for the causes of primate brain-size variation—both ecology and sociality appear to have influenced the evolution of brain size, but to different degrees in different clades. Each taxon has its own evolutionary history and ecology, constraints, and selection pressures. Our findings, as well as the overall results of previous studies (e.g. Table 1), reinforce the idea that as for locomotory, dietary, and physiological adaptations, primate brains are flexible and able to adapt to the challenges of their environment.

## Supplementary Material

Data available from the Dryad Digital Repository: http://dx.doi.org/10.5061/dryad.2547d7wpg. R scripts for the analysis are available at: https://github.com/mark-grabowski/brain_ms.
